# Comparative study of the modulation of fructose/sucrose-induced hepatic steatosis by mixed lipid formulations varying in unsaturated fatty acid content

**DOI:** 10.1186/s12986-015-0038-x

**Published:** 2015-11-14

**Authors:** Rafat A. Siddiqui, Zhidong Xu, Kevin A. Harvey, Thomas M. Pavlina, Michael J. Becker, Gary P. Zaloga

**Affiliations:** Methodist Research Institute, Indiana University Health, 1800 N. Capitol Ave, Indianapolis, IN 46202 USA; Baxter Healthcare Corporation, Deerfield, IL 60015 USA

**Keywords:** Hepatic steatosis, Non-alcoholic fatty liver disease (NAFLD), Nonalcoholic steatohepatitis (NASH), Parenteral-nutrition-associated liver disease (PNALD), Soybean oil, Olive oil, Macadamia nut oil, Fish oil, Lipid emulsion, Fatty acids, Liver, Sucrose, Fructose, Lipogenesis

## Abstract

**Background:**

Non-alcoholic fatty liver disease (NAFLD) is the most common cause of chronic liver disease in developed countries. NAFLD encompasses a spectrum of diseases, ranging from hepatic steatosis to non-alcoholic steatohepatitis (NASH), cirrhosis, and liver failure. The etiology of NAFLD remains unclear but is thought to relate to increased fatty acid flux within the liver that results in toxic fatty acid metabolite production. One source of increased fatty acid flux is fructose/sucrose-induced hepatic lipogenesis. Current treatment for NAFLD encompasses dietary modifications. However, little scientific evidence exists on which to base many dietary recommendations, especially the intake of different types of carbohydrates and fats. We hypothesized that lipid mixtures of unsaturated fatty acids would inhibit lipogenesis and subsequent hepatic steatosis induced by high carbohydrate diets. The aim of this study was to examine the effects of different complex mixtures of fatty acids upon the development of fructose/sucrose-induced hepatic steatosis.

**Methods:**

C57BL/6 mice were randomized to normocaloric chow-based diets that varied in the type of carbohydrate (starch, sucrose, fructose). Animals in each carbohydrate group were further randomized to diets that varied in lipid type (no additional lipid, soybean oil, fish oil, olive/soybean oil, macadamia nut oil). These oils were chosen based upon their content of omega-6 polyunsaturated fatty acids, omega-3 polyunsaturated fatty acids, omega-9 monounsaturated fatty acids, or omega-7 monounsaturated fatty acids. Fatty acid flux in the liver was determine by assessing hepatic lipid content (steatosis). We also assessed fatty acid levels in the plasma and liver of the animals, hepatic lipogenesis activity, hepatic stearoyl-CoA-1 desaturase activity, and hepatic elongase activity.

**Results:**

Animals consumed similar amounts of the diets and maintained normal body weights throughout the study. Both sucrose and fructose induced hepatic lipogenesis and steatosis, with fructose being more potent. All mixed lipids similarly inhibited steatosis, limiting lipid content to levels found in the control (starch) animals. Lipogenesis and stearoyl-CoA-1 desaturase activity were increased in the sucrose and fructose groups. Levels of these enzymatic processes remained at baseline in all of the lipid groups.

**Conclusion:**

This is the first study to compare various complex lipid mixtures, based upon dietary oils with different types of long-chain fatty acids, upon development of sucrose/fructose-induced steatosis. Both carbohydrate source and lipid content appear important for the modulation of steatosis. Moderate intake of complex lipids with high unsaturated to saturated fatty acid ratios inhibited both lipogenesis and steatosis.

## Background

Non-alcoholic fatty liver disease (NAFLD) is one of the most common forms of chronic liver disease in developed countries, affecting 20-60 % of the population [1–3]. NAFLD encompasses a spectrum of diseases that includes steatosis, nonalcoholic steatohepatitis (NASH), fibrosis, cirrhosis, liver failure, and hepatocellular carcinoma [1, 3, 4]. Development of steatosis and progression of steatosis to NASH and cirrhosis has been postulated to involve two insults [5, 6]. The first insult results in steatosis due to alterations in hepatic uptake, synthesis, metabolism, and secretion of fatty acids. The accumulation of lipids within hepatocytes predisposes the liver to a second insult that results in inflammation and oxidative injury. The net result is hepatic damage that progresses to fibrosis and liver failure. However, it is now believed that a more complex process is involved [4, 7, 8], which encompasses multiple parallel pathways that result in tissue injury and progression to fibrosis. Lipotoxicity represents one of the major metabolic pathways underlying hepatocyte dysfunction leading to disease progression [4, 7, 8]. It is postulated that excess free fatty acid (FFA) traffic (from diet, de novo lipogenesis, adipose lipolysis, impaired lipid oxidation) within hepatocytes that accompanies the development of steatosis leads to the generation of toxic lipid metabolites (i.e., acyl-CoA, lysophosphatidylcholine, ceramides, phosphatidic acid), which increase oxidative stress and inflammation (i.e., induction of NFκB, IL-6, IL-1, TNF-alpha), resulting in hepatocyte injury that leads to cirrhosis and liver failure [7, 9]. Increased fatty acid flux and triglyceride formation results largely from an increased supply of fatty acids (i.e., from lipolysis and diet) and de novo lipogenesis [10–12]. Both processes are increased in patients with NAFLD [10–12] and play important roles in lipid accumulation. Inhibition of hepatic steatosis (i.e., free fatty acid flux) has been shown to prevent progression of steatosis to NASH/cirrhosis [13–15].

The pathophysiologic role of diet in the development and progression of NAFLD is unclear. High caloric intake (overfeeding), high fat intake (especially saturated fatty acids), and high intake of simple sugars are all implicated in the pathogenesis of NAFLD. All of these dietary factors likely interact with genetic factors and result in hepatic lipid accumulation and cellular injury. Recent concern regarding NAFLD pathogenesis focuses upon high dietary intake of simple sugars [16–18], with fructose and sucrose being the most important sugars. Fructose/sucrose are used as sweeteners in many processed foods and beverages (especially soft drinks) [17, 18]. Fructose is known to stimulate de novo lipogenesis and inflammation/oxidation within the liver [18]. Although evidence is inconclusive, there is a growing belief that high fructose consumption is an important contributor to NAFLD [10, 17, 19, 20].

In addition to fructose/sucrose consumption, high fat intake and high saturated fatty acid intake [21, 22] have been implicated in the pathogenesis of NAFLD. In contrast to saturated fatty acids, high intake of polyunsaturated fatty acids reportedly inhibit NAFLD development [13, 23–25]. Polyunsaturated fatty acids also inhibit de novo lipogenesis [25–28]. In addition, fatty acids modulate inflammation, oxidation (including lipid peroxidation), immune reactions, endoplasmic reticulum stress, collagen formation, apoptosis and necrosis, endothelial integrity, and insulin resistance [4, 7]. Thus, the types of fatty acids that accumulate in hepatocytes during development of steatosis (from both endogenous and exogenous sources) are likely to modulate the development of NAFLD and its progression to NASH/cirrhosis.

We hypothesized that fructose interacts with lipid in the pathogenesis of NAFLD and that unsaturated fatty acids (i.e. oleic acid, palmitoleic acid, linoleic acid, docosahexaenoic acid, eicosapentaenoic acid) inhibit the development of steatosis. The goal of this study was to examine the effects of complex mixtures of fatty acids based upon different dietary oils (that reflects the types of lipids ingested by patients) upon the development of fructose/sucrose-induced hepatic steatosis. We compared the effects of mixtures of dietary lipids that varied in the quantities of omega-6 polyunsaturated fatty acids (soybean oil), omega-3 polyunsaturated fatty acids (fish oil), omega-9 monounsaturated fatty acids (olive oil, macadamia nut oil), and omega-7 monounsaturated fatty acids (macadamia nut oil) upon hepatic lipid content. This is the first study to compare the effects of these different mixed lipids, varying in unsaturated fatty acid type (i.e. oleic acid, palmitoleic acid, linoleic acid, docosahexaenoic acid, eicosapentaenoic acid), upon development of fructose/sucrose-induced hepatic steatosis.

## Methods

### Animals, study diets and lipid emulsions

C57BL/6 male mice, 6 weeks old, were housed under controlled temperature and humidity in the Methodist Research Institute’s Animal Care Facility, with a 12-hour light-dark cycle, and fed the study diets and water ad libitum. After 3 days of acclimation to the facility (receiving standard chow diets), mice were fed for the next 3 days with a low fat diet based on the AIN-76A rodent diet containing starch (corn starch and maltodextrin), sucrose, or fructose as the carbohydrate source. The starch diet was identical to the normal chow diet except for the reduced fat content. The diets provided similar quantities of carbohydrate (665 g/kg), protein (casein; 200 g/kg), and lipid (soybean oil; 5 g/kg). A small amount of soybean oil was added to the diets to meet essential fatty acid requirements of the animals. The diets were supplemented with recommended amounts of DL-methionine (3 g/kg), vitamins, and minerals. In preliminary studies (not published), we established that the sucrose and fructose diets induced hepatic steatosis in the animals. The study lipids were provided in the form of a lipid emulsion and fed to animals for days 3-8 of the study. In previous studies, we found that supplying the oils as an emulsion improved absorption and none of the animals developed loose stools or diarrhea.

In this study, animals were randomly divided into three groups that received starch (normal diet), sucrose, or fructose as the carbohydrate source; each carbohydrate group was further randomized into five groups that received no additional lipid, or one of the four lipid emulsions for a total of 15 groups of 5 animals per group. Lipid emulsions were administered to animals daily (day 3-8) by gavage and supplied 90 mg/day of lipid to the animals. Lipid emulsion triglyceride concentrations are reported as percent (%) which reflects weight/volume (g/dL) of lipid. Lipid consisted of a soybean oil emulsion that was high in omega-6 polyunsaturated fatty acids (SO; 450 μl/day of a 20 % or 20 g/dL emulsion; Intralipid, Fresenius Kabi, Bad Homburg, Germany), an 80 % olive oil and 20 % soybean oil emulsion that was high in omega-9 monounsaturated fatty acids (OOSO; 450 μl/day of a 20 % or 20 g/dL emulsion; ClinOleic, Baxter Healthcare, Deerfield, Illinois), a macadamia nut oil emulsion that was high in omega-7 and omega-9 monounsaturated fatty acids (MNO; 450 μl/day of a 20 % or 20 g/dL emulsion; Baxter Healthcare, Deerfield, Illinois), and a fish oil emulsion high in omega-3 polyunsaturated fatty acids (FO; 900 μl/day of a 10 % or 10 g/dL emulsion; Omegaven, Fresenius Kabi, Bad Homburg, Germany). The FO emulsion was only available as a 10 % lipid emulsion while the other three emulsions were available as 20 % emulsions. All lipid emulsions contained 1.2 g/dL of egg yolk phospholipids.

Body weight was recorded daily. Blood (for plasma) and liver tissues were collected at the end of the study and stored at −80 °C until analyzed. The protocol for these studies was approved (protocol # 2010-17) by the Methodist Research Institute’s Animal Research Committee (Animal Welfare Assurance Number-A3772-010) and strictly followed the *Guide for the Care and Use of Laboratory Animals* (NIH publication No.85-23, revised 1996)*.*

### Fatty acid analysis

Lipid emulsions (20 μL each) did not require fatty acid extraction prior to analysis; therefore, these samples were directly transmethylated in conjunction with an internal standard (tricosanoic acid). Methanol-benzene 4:1 and acetyl chloride were added to the samples while on dry ice. The samples were placed in tubes, purged with nitrogen gas, and subjected to methanolysis at room temperature for 24 h. The reaction was stopped and neutralized with K_2_CO_3_. Layers were separated by centrifugation and an aliquot of the benzene layer was removed for gas chromatography analysis [29]. For liver analysis of fatty acids, a small sample (approximately 200 mg) was removed from an −80 °C freezer, thawed on ice, and homogenized in cold PBS. An internal standard of tricosanoic acid was added to the homogenates and methanol:benzene (4:1; v:v) added. Acetyl chloride was added while on dry ice, the samples sealed, transferred to room temperature, and maintained until the reaction mixture was melted. Transmethylation was performed as described above. Fatty acid extraction of the mouse plasma (100 μL) prior to analysis was not required; therefore, direct transmethylation of these samples were performed as described above.

For gas chromatography analysis of fatty acids, the benzene layer was recovered following centrifugation and fatty acids were separated on a gas chromatography system (Shimadzu GC2010) equipped with a Zebron ZB-WAX plus column (100 m, 0.25 mm ID, 0.25 μm film thickness) as described previously [29]. The oven temperature was programmed from 30 °C (2 min hold) to 180 °C @ 20 °C/min (2 min hold), then to 207 °C @ 4 °C/min (3 min hold), then to 220 °C @ 2 °C/min (2 min hold), and finally to 240 °C @ 2 °C/min (2 min hold). Detection was performed with a FID @ 250 °C to resolve fatty acid peaks, which were identified using authentic standards (Restek Corp., Bellefonte, PA). Data were analyzed with Shimadzu's GC solutions software. Data are presented either as mg or μg/volume or weight of tissues.

### Oil Red O staining for lipid droplets

The liver tissues, which were frozen in OCT compounds, were cut into 5 μm sections using a cryostat (Leica CM1900; Leica Microsystems, Bannockburn, IL, USA). The sections were mounted on slides and allowed to dry for 1-2 h. The sections were fixed with 10 % formalin for 10 min, and then the slides were rinsed with PBS (PH 7.4). After air dry, the slides were placed in 85 % propylene glycol for 2 min, and stained in 0.5 % Oil Red O solution in propylene glycol for 30 min. The slides were transferred to an 85 % propylene glycol solution for 1 min, rinsed in distilled water for 2 changes, and processed for hematoxylin counter staining.

### Triglyceride levels in hepatic tissues

Analysis of triglyceride in liver tissues was performed using a commercial kit (Cayman Chemicals, MI). Briefly liver tissues (100-300 mg) were homogenized in 2 ml of standard diluent containing a mixture of protease inhibitor cocktail (Roach Diagnostics, IN). The samples were centrifuged at 10,000 × g for 10 min at 4 °C and the supernatant was used to determine triglycerides as per manufacturer’s instructions.

### Calculation of fatty acid synthesis enzyme activity

The ratio of 16:0 to 18:2n-6 was used to calculate a de novo lipogenesis (DNL) index [30, 31]. The ratio of 18:1n-9/18:0 was used to calculate hepatic stearoyl- CoA-1 (delta-9) desaturase activity [23, 32]. The ratio of 18:0/16:0 was used to calculate elongase activity [33].

### Statistical analysis

The data are reported as mean ± SD unless stated otherwise. All comparisons are made by one-way ANOVA with Tukey’s post hoc test using SPSS Statistics 20 software except for individual fatty acid analyses. Fatty acids analyses were done on the normalized data (not adjusted for interdependence) using “R (version 1.15.1)” software [34]. Means differences were compared using studentized range with Tukey’s hsd (honestly significant difference). All significant values are reported at *P* <0.05. Values with different letters or symbols are significantly different from each other.

## Results

### Fatty acid composition of lipid emulsions

We selected four different lipid emulsions for study based on their content of n-3, n-6, n-7, and n-9 long-chain fatty acids (Table 1). The SO emulsion contained linoleic acid (n-6 polyunsaturated fatty acid) as the major fatty acid, accounting for nearly 56 % of total fatty acid content. The OOSO emulsion contained oleic acid (n-9 monounsaturated fatty acid) as the major fatty acid (approximately 59 % of the total fatty acid content). The MNO emulsion contained approximately 74 % monounsaturated fatty acids, of which 21 % of total fatty acids was the n-7 monounsaturated fatty acid palmitoleic acid (C16:1n-7) and 52 % was the n-9 monounsaturated fatty acid oleic acid (C18:1n-9). This oil is one of the highest dietary sources of n-7 monounsaturated fatty acids. The MNO lipid had the lowest amount of polyunsaturated fatty acids, accounting for only 4 % of total fatty acids. The FO emulsion contained approximately 50 % of fatty acids as long-chain n-3 polyunsaturated fatty acids with approximately a 1:1 eicosapentaenoic acid (EPA) to docosahexaenoic acid (DHA) ratio. All lipid emulsions contained similar amounts of saturated fatty acids, thus accounting for approximately 15-17 % (mean values) of total fatty acids. The lipid emulsions differed in the ratio of polyunsaturated to saturated fatty acids and monounsaturated to saturated fatty acids. However, they had similar unsaturated to saturated fatty acid ratios.

**Table 1:** Fatty acid composition of lipid emulsions. Values (mean ± SD) represent % of total fatty acids

*n* = 5	SO	OOSO	MNO	FO
n=5	%	%	%	%
14:0	0.04 ± 0.002		0.63 ± 0.002	4.28 ± 0.02
c16:0	11.03 ± 0.1	12.66 ± 0.03	9.03 ± 0.03	10.05 ± 0.18
16:1n-7	0.08 ± 0.001	0.98 ± 0.004	**21.04 ± 0.04**	7.92 ± 0.03
16:2n-4				1.20 ± 0.03
16:3n-4				1.58 ± 0.013
18:0	3.88 ± 0.03	3.56 ± 0.009	2.88 ± 0.02	2.24 ± 0.21
18:1n-9	20.72 ± 0.14	**59.13 ± 0.79**	**53.23 ± 0.17**	9.15 ± 0.13
18:1n-7	1.52 ± 0.03	2.20 ± 0.02	3.19 ± 0.13	2.43 ± 0.04
18:2n-6	**55.61 ± 0.39**	18.18 ± 0.02	3.25 ± 0.005	2.69 ± 0.007
18:3n-6				0.24 ± 0.006
18:3n-3	5.69 ± 0.04	2.10 ± 0.003	0.13 ± 0.002	0.96 ± 0.01
18:4n-3				4.03 ± 0.01
20:0	0.29 ± 0.002	0.34 ± 0.001	2.11 ± 0.005	
20:1n-9	0.19 ± 004	0.21 ± 0.003	2.78 ± 0.05	0.85 ± 0.02
20:4n-6	0.18 ± 0.002	0.18 ± 0.001	0.19 ± 00.002	1.47 ± 0.03
20:4n-3				1.03 ± 0.004
20:5n-3				**21.73 ± 0.03**
22:0	0.35 ± 0.002	0.12 ± 0.001	0.70 ± 0.003	
22:1n-9			0.33 ± 0.006	
22:5n-6				0.467 ± 0.010
22:5n-3				2.12 ± 0.01
22:6n-3	0.14 ± 0.002	0.08 ± 0.004	0.04 ± 0.001	**20.63 ± 0.20**
Total	100 ± 0.73	100 ± 0.91	100 ± 0.359	100 ± 0.846
Total SFA	15.59 ± 0.13	16.68 ± 0.04	15.35 ± 0.08	17.02 ± 0.41
Total MUFA	22.51 ± 0.02	62.53 ± 0.85	80.57 ± 0.28	20.35 ± 0.21
n-7	1.60 ± 0.03	3.18 ± 0.03	24.23 ± 0.05	10.35 ± 0.07
n-9	20.19 ± 0.15	59.35 ± 0.82	56.34 ± 0.23	10.00 ± 0.15
Total PUFA	61.63 ± 0.44	20.54 ± 0.03	3.91 ± 0.01	57.12 ± 0.39
n-3	5.83 ± 0.04	2.18 ± 0.01	0.17 ± 0.003	49.47 ± 0.30
n-6	55.80 ± 0.40	18.36 ± 0.02	3.74 ± 0.01	4.87 ± 0.05
PUFA/SFA	4.0	1.2	0.25	3.4
MUFA/SFA	1.4	3.7	5.2	1.2
USAT/SFA	5.4	5.0	5.5	4.6

*SO* soybean oil (20 g/dl), *OOSO*olive oil/soybean oil (20 g/dl), *MNO* macadamia nut oil (20 g/dl), *FO* fish oil (10 g/dl), *PUFA* polyunsaturated fatty acids, *MUFA* monounsaturated fatty acids, *SFA* saturated fatty acids, *USAT* unsaturated fatty acids

### Body weights and food intake

**Fig. 1:**
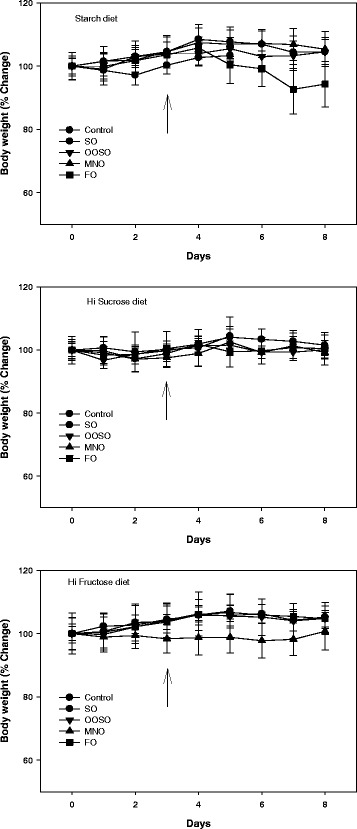
Effects of study diets upon changes in body weight over time. Mice in test groups were fed for 3 days with a low-fat diet containing starch, sucrose, or fructose as the carbohydrate source. The study lipids were provided in the form of a lipid emulsion and fed to animals for days 3-8 of the study. The arrows indicate start of lipid emulsion feeding. Values are mean ± SD of 5 animals in each group. No statistical difference is found between emulsion treatments in each group

The daily body weights of animals (mean ± SD) on the study diets (i.e., starch, sucrose, and fructose) were not significantly different from each other before (starch, 21.80 ± 0.77 g; sucrose, 21.38 ± 0.44 g; fructose, 21.62 ± 0.82 g) or after (starch, 22.26 ± 01.16 g; sucrose, 21.73 ± 0.70 g; fructose, 22.45 ± 0.92 g) lipid emulsion administration (Fig. 1). None of the animals were considered obese. We also recorded average food intake by animals in each group, and there were no significant differences in food intake (2.5-3.0 g/day) among the different dietary groups and among oil emulsion treatments. Overall, the animals maintained their body weight during the study, and there were no significant differences among control (no additional lipid) and lipid emulsion groups for each diet. However, there was a trend for lower body weight in the fish oil starch diet and macadamia fructose diet groups.

### Effect of diets and lipid emulsions on plasma fatty acid composition in mice fed high carbohydrate diets

**Fig. 2:**
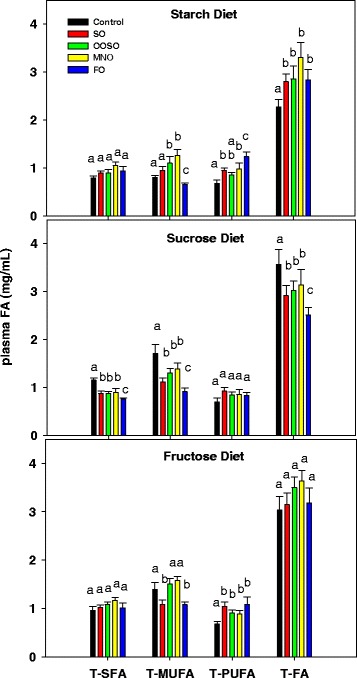
Plasma fatty acids levels (mg/ml) in animals receiving the starch, sucrose, and fructose diets. Total saturated fatty acids (T-SFA), total monounsaturated fatty acids (T-MUFA), total polyunsaturated fatty acids (T-PUFA) and total fatty acids (T-FA) in plasma. Controls received the low-fat, high-carbohydrate diets with no added lipid. Values are mean ± SD of 5 animals in each group. Different letters indicate that groups are significantly different at *P* <0.05. Treatments with the same letter are not significantly different from each other

The sucrose diet increased saturated (16:0, 18:0) and monounsaturated fatty acids (18:1n-7, 18:1n-9) in the plasma (Table 2, Fig. 2) compared to the starch diet group (control). The fructose diet did not significantly alter plasma fatty acids compared to the starch diet group. The SO, OOSO, and MNO lipids (Table 2) had little effect upon plasma fatty acids. However, plasma n-3 polyunsaturated fatty acids were increased by FO. Total plasma fatty acids were increased by all lipid emulsions in the starch diet group (Fig. 2), decreased in the sucrose diet group, and similar to the no added lipid control group in the fructose diet group. Plasma saturated fatty acids were decreased by all lipid emulsions in the sucrose group but not in the fructose group (Fig. 2). Monounsaturated fatty acids were decreased by all lipid emulsions in the sucrose group, but only by SO and FO in the fructose group.

**Table 2:** Plasma fatty acids (mg/ml; mean ± SD) of animals fed high carbohydrate diets

Fatty acids^∆^	Control (no added lipid)	SO	OOSO	MNO	FO
A. Starch diet					
16:0	0.57 ± 0.07^a^	0.66 ± 0.07	0.67 ± 0.14	0.77 ± 0.09	0.62 ± 0.06
16:1n-7	0.15 ± 0.02^a^*†	0.17 ± 0.04*	0.21 ± 0.08*	0.22 ± 0.05*	0.11 ± 0.02†
18:0	0.17 ± 0.01^a^	0.19 ± 0.03	0.18 ± 0.02	0.23 ± 0.04	0.25 ± 0.08
18:1n-9	0.51 ± 0.02^a^*†	0.62 ± 0.10*†	0.71 ± 0.18*†	0.83 ± 0.15*	0.47 ± 0.02†
18:1n-7	0.12 ± 0.02^a^	0.13 ± 0.03	0.15 ± 0.05	0.17 ± 0.05	0.07 ± 0.01
18:2n-6	0.35 ± 0.05^a^*†	0.50 ± 0.05*	0.45 ± 0.06*	0.46 ± 0.08*	0.37 ± 0.02†
20:4n-6	0.19 ± 0.05^a^	0.28 ± 0.02	0.24 ± 0.02	0.30 ± 0.06	0.22 ± 0.05
20:5n-3	<0.01	<0.01	<0.01	<0.01	0.24 ± 0.03*
22:6n-3	0.09 ± 0.02^a^	0.13 ± 0.02	0.13 ± 0.02	0.12 ± 0.07	0.33 ± 0.09*
B. Sucrose diet					
16:0	0.86 ± 0.05^b^	0.60 ± 0.09	0.62 ± 0.06	0.63 ± 0.11	0.53 ± 0.02
16:1n-7	0.21 ± 0.04^a^	0.16 ± 0.01	0.21 ± 0.02	0.21 ± 0.07	0.17 ± 0.03
18:0	0.24 ± 0.02^b^	0.22 ± 0.03	0.19 ± 0.02	0.21 ± 0.03	0.18 ± 0.01
18:1n-9	1.21 ± 0.23^b^	0.73 ± 0.13*,†	0.84 ± 0.12*,†	0.90 ± 0.13*	0.61 ± 0.10†
18:1n-7	0.24 ± 0.04^b^	0.18 ± 0.03	0.21 ± 0.04	0.22 ± 0.01	0.13 ± 0.03
18:2n-6	0.31 ± 0.04^a^†	0.41 ± 0.07*	0.41 ± 0.04*	0.36 ± 0.07*†	0.22 ± 0.03§
20:4n-6	0.22 ± 0.05^a^	0.28 ± 0.05	0.27 ± 0.04	0.29 ± 0.06	0.16 ± 0.02*
20:5n-3	<0.01	<0.01	<0.01	<0.01	0.16 ± 0.03*
22:6n-3	0.11 ± 0.02^a^	0.14 ± 0.02	0.13 ± 0.02	0.14 ± 0.02	0.24 ± 0.03*
C. Fructose diet					
16:0	0.54 ± 0.32^a^	0.71 ± 0.09	0.79 ± 0.08	0.69 ± 0.40	0.74 ± 0.15
16:1n-7	0.12 ± 0.07^a^	0.17 ± 0.05	0.24 ± 0.05	0.22 ± 0.13	0.17 ± 0.03
18:0	0.19 ± 0.11^a,b^	0.27 ± 0.02	0.23 ± 0.01	0.20 ± 0.11	0.22 ± 0.04
18:1n-9	0.80 ± 0.47^a,b^	0.70 ± 0.11	0.97 ± 0.13	0.80 ± 0.45	0.78 ± 0.07
18:1n-7	0.15 ± 0.09^a,b^	0.16 ± 0.04	0.24 ± 0.03*	0.20 ± 0.12	0.02 ± 0.02†
18:2n-6	0.20 ± 0.12^a^	0.50 ± 0.12	0.42 ± 0.04	0.35 ± 0.21	0.27 ± 0.06
20:4n-6	0.18 ± 0.10^a^	0.33 ± 0.05	0.29 ± 0.04	0.21 ± 0.12	0.22 ± 0.06
20:5n-3	<0.01	<0.01	<0.01	<0.01	0.19 ± 0.05*
22:6n-3	0.08 ± 0.05^a^	0.16 ± 0.03	0.14 ± 0.01	0.12 ± 0.07	0.33 ± 0.09*

^∆^Fatty acids with levels greater than 1 % of total fatty acids. Different alphabets (a, b) represent a statistical difference at *P* <0.05 between carbohydrate diets (no added lipids) whereas different symbols (*,†,§) represent a statistical difference between control and emulsion groups within each carbohydrate dietary group. Groups with the same alphabet letters or symbols are not statistically different from each other

### Effect of diets and lipid emulsions on hepatic fatty acid composition in mice fed high carbohydrate diets

**Fig. 3:**
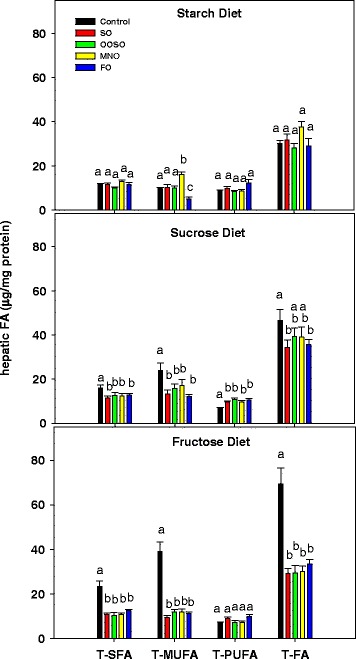
Hepatic fatty acid levels (μg/mg protein) in animals receiving the starch, sucrose, and fructose diets. Total saturated fatty acids (T-SFA), total monounsaturated fatty acids (T-MUFA), total polyunsaturated fatty acids (T-PUFA) and total fatty acids (T-FA) in liver. Controls received the low fat high carbohydrate diets with no added lipid. Values are mean ± SD of 5 animals in each group. Different letters indicate that groups are significantly different at *P* <0.05. Treatments with the same letter are not significantly different from each other

The sucrose diet increased total hepatic fatty acids by 57 % over levels in starch control animals. The higher fat content resulted from increases in saturated (16:0) and monounsaturated fatty acids (16:1n-7, 18:1n-7, 18:1n-9) (Table 3, Fig. 3). Fructose increased total hepatic fatty acids by 133 % by increasing both saturated (16:0) and monounsaturated fatty acid levels (16:1n-7, 18:1n-7, 18:1n-9).

**Table 3:** Hepatic fatty acids (μg/mg protein; mean ± SD) of animals fed high carbohydrate diets

Fatty acids^∆^	Control (no added lipid)	SO	OOSO	MNO	FO
A. Starch diet					
16:0	7.59 ± 0.73^a^	7.84 ± 1.06	6.64 ± 0.53	9.26 ± 0.99	6.80 ± 0.61
16:1n-7	1.53 ± 0.15^a^	1.53 ± 0.57	1.46 ± 0.30	2.26 ± 0.48	0.61 ± 0.37*
18:0	3.42 ± 0.16^a^	3.26 ± 0.14	2.80 ± 0.30	3.16 ± 0.14	4.43 ± 0.92
18:1n-9	5.91 ± 0.63^a^	6.68 ± 1.77	6.49 ± 1.06	10.58 ± 1.48*	3.65 ± 1.05†
18:1n-7	1.74 ± 0.41^a^	1.61 ± 0.46	1.60 ± 0.51	2.66 ± 0.48	0.70 ± 0.14*
18:2n-6	3.14 ± 0.09^a^	3.89 ± 0.88	3.27 ± 0.44	3.00 ± 0.64	3.28 ± 1.45
20:4n-6	3.50 ± 0.23^a^	3.62 ± 0.30	3.06 ± 0.47	3.22 ± 0.32	3.05 ± 0.60
20:5n-3	0.03 ± 0.02^a^	0.02 ± 0.02	0.01 ± 0.01	0.01 ± 0.02	1.23 ± 0.50*
22:6n-3	2.01 ± 0.13^a^	2.22 ± 0.24	1.84 ± 0.18	2.00 ± 0.29	4.38 ± 0.15*
B. Sucrose diet					
16:0	12.06 ± 1.47^b^	7.25 ± 1.25	8.29 ± 1.56	8.06 ± 1.60	8.23 ± 0.81
16:1n-7	2.44 ± 0.41^b^	1.27 ± 0.23	1.99 ± 0.50	1.95 ± 0.57	1.70 ± 0.14
18:0	3.31 ± 0.61^a^	3.78 ± 0.36	3.87 ± 0.52	3.67 ± 0.17	4.02 ± 0.33
18:1n-9	17.34 ± 4.92^b^	8.98 ± 2.72*	10.22 ± 2.65*	11.26 ± 3.61*	8.30 ± 1.43*
18:1n-7	3.38 ± 0.29^b^	2.34 ± 0.76	2.82 ± 0.63	3.08 ± 0.38	1.92 ± 0.24
18:2n-6	2.03 ± 0.06^b^	3.32 ± 0.59	3.86 ± 0.72	3.17 ± 0.45	2.36 ± 0.42
20:4n-6	2.80 ± 0.19^a^	3.92 ± 0.37	4.15 ± 0.34	3.86 ± 0.28	2.68 ± 0.22
20:5n-3	0.01 ± 0.02^a^	0.03 ± 0.01	0.04 ± 0.02	0.02 ± 0.02	1.41 ± 0.21*
22:6n-3	1.55 ± 0.18^a^	2.07 ± 0.23*	2.46 ± 0.18*	2.25 ± 0.30*	3.93 ± 0.34†
C. Fructose diet					
16:0	18.27 ± 4.35^c^	6.83 ± 1.20*	6.84 ± 1.95*	7.24 ± 1.20*	8.27 ± 0.50*
16:1n-7	3.30 ± 1.29^b^	1.08 ± 0.40*	1.38 ± 0.46*	1.58 ± 0.42*	1.36 ± 0.12*
18:0	4.24 ± 0.25^a^	3.59 ± 0.30	3.12 ± 0.48	3.06 ± 0.24	3.71 ± 0.33
18:1n-9	29.43 ± 5.86^c^	6.05 ± 1.15*	7.94 ± 1.32*	7.58 ± 2.17	8.10 ± 0.73*
18:1n-7	5.10 ± 1.25^c^	1.71 ± 0.53*	2.05 ± 0.51*	2.26 ± 0.41*	1.49 ± 0.29*
18:2n-6	2.03 ± 0.40^b^	3.23 ± 0.88	2.29 ± 0.61	2.49 ± 0.52*	1.90 ± 0.54
20:4n-6	2.68 ± 0.17^a^	3.51 ± 0.19	3.04 ± 0.59	2.92 ± 0.24	2.53 ± 0.29
20:5n-3	<0.01^a^	0.01 ± 0.02	0.01 ± 0.01	0.01 ± 0.01	1.17 ± 0.22*
22:6n-3	1.44 ± 0.21^a^	2.03 ± 0.33	1.59 ± 0.37	1.75 ± 0.27	3.95 ± 0.55*

^∆^Fatty acids with levels greater than 1 % of total fatty acids. Different alphabets represent a statistical difference at *P* <0.05 between diets (no added lipids) whereas different symbols represent a statistical difference between emulsion groups within each dietary group

The effects of the lipid emulsions upon hepatic fatty acid content in the starch group reflected the composition of the lipid emulsions (Table 3, Fig. 3). FO elevated n-3 polyunsaturated fatty acids (20:5n-3, 22:6n-3) and decreased monounsaturated fatty acids (16:1n-7, 18:1n-7, 18:1n-9). MNO increased monounsaturated fatty acids (18:1n-9).

All four lipid emulsions decreased total, saturated, and monounsaturated fatty acid concentrations in the sucrose and fructose groups by comparable amounts. Fatty acid levels were reduced to values found in the starch group (Fig. 3). In addition, FO significantly increased n-3 polyunsaturated fatty acids and SO maintained higher n-6 polyunsaturated fatty acid levels.

### Effect of lipid emulsions on hepatic fat content in mice fed high carbohydrate diets

**Fig. 4:**
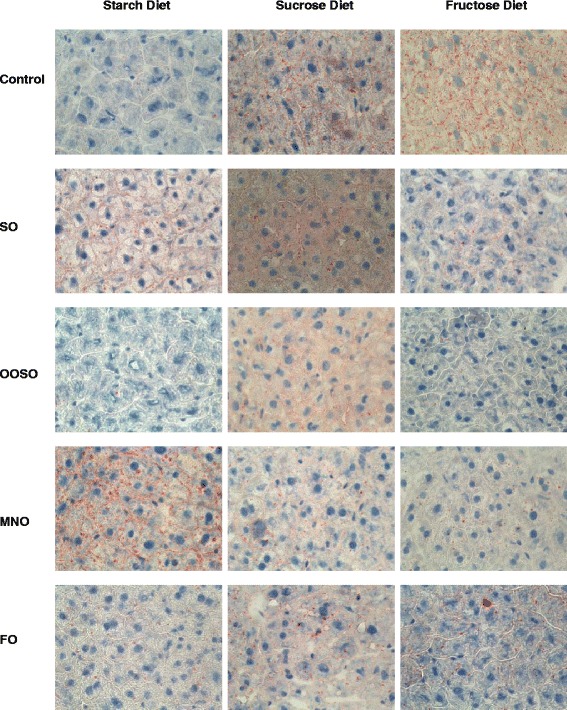
Accumulation of lipid droplets in liver from animals receiving the starch, sucrose, and fructose diets. The liver tissue sections were first stained with Oil Red O solution and then counter stained with hematoxylin. Controls received the low fat high carbohydrate diets with no added lipid. Each picture (400x) is a representative of 5 liver sections from each group

**Fig. 5:**
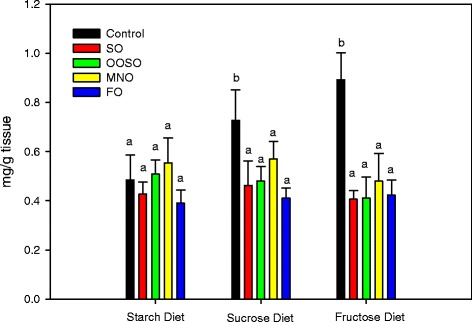
Triglyceride levels (mg/g tissue) in the livers of animals receiving the starch, sucrose, and fructose diets. Controls received the low-fat, high-carbohydrate diets with no added lipid. Values are mean ± SD of 5 animals in each group. Different letters indicate that groups are significantly different at *P* <0.05. Treatments with the same letter are not significantly different from each other

We further explored fat accumulation in hepatic tissues by assaying tissue samples for lipid droplet accumulation (Oil Red O staining) and total triglyceride levels. Compared to animals on the starch diet, there was increased accumulation of lipid droplets in animals on the sucrose and fructose diets as evidenced by increased staining (Fig. 4). Fructose induced a greater number of droplets than sucrose. Treatment of animals receiving the starch diet with SO or MNO resulted in lipid droplet accumulation; the OOSO and FO diets induced minimal lipid droplet accumulation. Administration of all four lipid emulsions to mice receiving the sucrose and fructose diets reduced lipid droplet formation. We further validated this data by measuring total triglyceride levels in liver samples. Consistent with lipid droplet accumulation, data shown in Fig. 5 indicate that the sucrose and fructose diets resulted in increased triglyceride levels in the liver compared to the starch diet group. Treatment with lipid emulsions reduced sucrose- and fructose-induced triglyceride accumulation to levels seen in the starch diet group. The effect was independent of type of lipid emulsion used.

### Effect of diet and emulsion on lipid metabolism

**Fig. 6:**
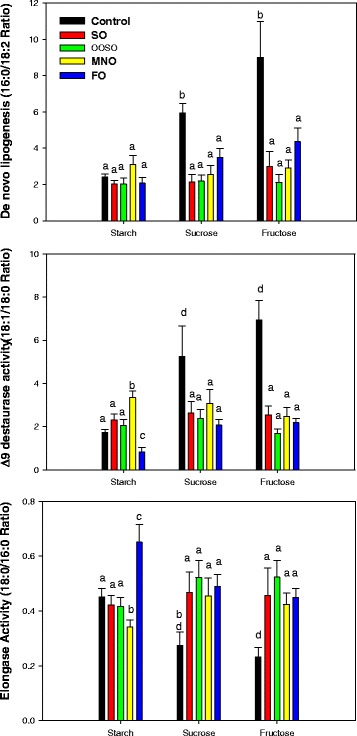
Effect of diet (starch, sucrose, and fructose) upon liver de novo lipogenesis, delta-9 desaturase activity, and elongase activity. Controls received the low-fat, high-carbohydrate diets with no added lipid. Values are mean ± SD of 5 animals in each group. Different letters indicate that groups are significantly different at *P* <0.05. Treatments with the same letter are not significantly different from each other

Hepatic de novo lipogenesis, as assessed using the 16:0/18:2 index, was increased significantly in both sucrose (2.5-fold) and fructose (3.5-fold) diet control groups (Fig. 6). Treatment with lipid emulsions significantly reduced the sucrose and fructose induced lipogenesis indices to values similar to those in the starch diet group. The effect was independent of the lipid emulsion composition. Feeding sucrose and fructose also increased delta-9 desaturase activity (approximately 3- to 4-fold) compared to animals on the starch diet (Fig. 6). Treatment with MNO caused a small but significant increase, whereas treatment with FO caused a significant decrease in delta-9 desaturase activity in livers from the starch fed diet group. Treatment of the sucrose/fructose groups with all lipid emulsions decreased delta-9 desaturase activity to levels found in the starch diet group. Interestingly, sucrose and fructose caused a significant reduction in elongase activity compared to that of starch fed animals (Fig. 6). Treatment with FO caused a significant increase in elongase activity in livers of starch fed animals. Treatment of the sucrose and fructose diet groups with lipid emulsion increased elongase activity to values found in the starch diet animals.

## Discussion

NAFLD represents a group of diseases that result from increased fatty acid flux within the liver. The most common manifestation of NAFLD is hepatic steatosis. However, NAFLD may progress to NASH, cirrhosis, and liver failure. NAFLD is associated with increased morbidity, mortality, and medical costs [2, 4, 11, 12]. Treatment of NAFLD is based upon weight reduction and exercise in overweight patients and dietary modifications designed to reduce hepatic fatty acid fluxes. However, scientific evidence on which to base dietary recommendations to reduce hepatic lipid are lacking. The aim of this study was to evaluate the effects of different dietary fatty acid mixtures upon the development of hepatic steatosis.

### Effect of sucrose/fructose upon development of hepatic steatosis

In this study, we evaluated the effects of different carbohydrate sources (i.e., starch, sucrose, and fructose) upon the development of hepatic steatosis. Our findings that both sugar diets (fructose and sucrose) significantly increased hepatic lipid content (steatosis) above levels found in the starch diet (control) group, with the fructose diet being more potent than the sucrose diet, are consistent with other studies [8, 19, 20, 23].

### Effect of lipid mixtures upon development of hepatic steatosis

We evaluated the effects of complex lipid mixtures (based upon different dietary oils) that varied in their content of n-3 and n-6 polyunsaturated fatty acids, n-7 and n-9 monounsaturated fatty acids, and the polyunsaturated/saturated fatty acid ratio upon the development of sucrose/fructose induced hepatic steatosis. The main finding of the study is that different mixtures of unsaturated fatty acids (i.e. oleic acid, palmitoleic acid, linoleic acid, eicosapentaenoic acid, docosahexaenoic acid) similarly inhibited the development of sucrose/fructose induced hepatic steatosis. Neither the specific quantity of polyunsaturated (i.e. linoleic acid, eicosapentaenoic acid, docosahexaenoic acid) or monounsaturated fatty acids (i.e. oleic acid, palmitoleic acid) nor the ratio of polyunsaturated to saturated fatty acids (varying from 0.25 to 4.0) affected the inhibitory results. Steatosis was also independent of the n-6 polyunsaturated/n-3 polyunsaturated fatty acid ratio of the lipid mixtures, and emulsions containing predominantly n-3 polyunsaturated fatty acids (i.e. eicosapentaenoic acid, docosahexaenoic acid), n-6 polyunsaturated fatty acids (i.e. linoleic acid), or n-7/n-9 monounsaturated fatty acids (i.e. oleic acid, palmitoleic acid) were equally effective at inhibiting steatosis. However, the lipid mixtures all had similar unsaturated/saturated fatty acid ratios (varying from 4.6 to 5.5). All of the study lipid mixtures inhibited the development of fructose/sucrose-induced hepatic steatosis, reducing levels to those found in the starch control diet group. The lipid emulsions had no effect on hepatic lipid content in the starch control group. Thus, the inhibitory effect depended upon induction of lipogenesis. It is important to note that the lipid mixtures used in this study had high unsaturated/saturated fatty acid ratios (an attribute of the oils used to make the lipid emulsions). In addition, saturated fatty acid content (predominantly palmitic acid and stearic acid) was approximately 16 % across all the lipid emulsion mixtures. It is unclear if lipid mixtures with lower unsaturated to saturated fatty acid ratios or higher saturated fatty acid contents would have similar effects.

### Mechanisms for lipid induced inhibition of hepatic steatosis

The increase in hepatic lipid content in the sucrose/fructose diet groups was in the form of triglycerides and primarily reflected increases in saturated and monounsaturated fatty acids, consistent with increased hepatic de novo lipogenesis. In addition, we found that the fructose/sucrose diets increased de novo lipogenesis activity and delta-9 desaturase activity. Excess carbohydrate intakes, particularly in the form of sucrose or fructose, are known to stimulate de novo lipogenesis [8, 10, 17]. The increase in lipogenesis found in this study is comparable to the increased lipogenesis found in patients with NAFLD [10–12]. Thus, our study results suggest that the primary mechanism for the lipid inhibitory effects upon steatosis relates to inhibition of lipogenesis induced by the sugars. Total polyunsaturated fatty acid content of the liver was not altered by the sucrose/fructose diets. Polyunsaturated fatty acids reflect dietary intake (essential fatty acids) and adipose tissue lipolysis (from stored dietary polyunsaturated fatty acids), rather than de novo lipogenesis. The lack of effect of the lipid emulsions upon liver lipid content in the starch control group likely reflects the low level of hepatic lipid and low lipogenesis activity in this group.

The stimulation of lipogenesis by high carbohydrate intakes results from activation of transcription factors (i.e. steroid responsive element binding protein 1c; SREBP1c) and increased expression of lipogenic enzymes (i.e. fatty acid synthase [FAS], stearoyl-CoA desaturase, acetyl-CoA carboxylase [ACC], malic enzyme) [8, 10, 17]. In contrast to carbohydrate, many fatty acids (i.e. n-3 and n-6 polyunsaturated fatty acids) suppress lipogenesis by inhibiting SREBP1c and transcription of genes involved in lipogenesis [23, 25–28]. Additionally, some fatty acids (i.e. n-3 polyunsaturated fatty acids) have been found to upregulate expression of peroxisomal proliferator-activated receptor alpha (PPARα) which increases fatty acid degradation [13, 25]. Thus, fatty acids can increase fatty acid degradation and decrease fatty acid synthesis. The net result would be decreased storage of lipids in tissues (i.e. less hepatic steatosis).

Many studies have shown that both n-6 and n-3 polyunsaturated fatty acids suppress lipogenesis and development of hepatic steatosis [13, 23, 25–28, 35–37]. However, most studies using dietary oils failed to directly compare n-6 with n-3 polyunsaturated fatty acids. Thus, it is difficult to compare the potency of oils containing different quantities of these polyunsaturated fatty acid mixtures. In contrast, saturated fatty acids have been reported to have little inhibitory effects upon lipogenesis [13, 26–28, 36] and high fat diets enriched with saturated fatty acids [23, 25, 38, 39] have been reported to increase lipogenesis and hepatic steatosis.

A limited number of studies have evaluated the effects of monounsaturated fatty acids or oils containing high monounsaturated fatty acids upon hepatic lipid metabolism such as lipogenesis and hepatic steatosis. The effects of olive oil upon hepatic steatosis have been variable with reports of increased, decreased, or no effect upon steatosis [40, 41]. The variability appears to depend upon the model used, amount and type of olive oil, quality and composition of the olive oil, control group used for comparison, and the fat/calorie content of the diets. Potential mechanisms for decreased steatosis with olive oil include decreased hepatic expression of lipogenic genes through inhibition of SREBP1c, increased hepatic fatty acid oxidation through stimulation of PPARα, increased secretion of very low density lipoproteins, anti-inflammatory effects, and anti-oxidant actions [40, 41]. Our results using olive oil are comparable to previous studies using different animal models [40–43]. For example, Wani et al. [42] induced hepatic steatosis in mice using a high fat diet (42 % of energy as fat; over 16 weeks). Administration of olive oil with the high fat diet decreased weight gain, serum triglycerides, normalized liver enzyme levels, and decreased hepatic steatosis. Deng et al. [43] utilized JCR:LA-cp rats (lack leptin receptors) to study hepatic steatosis. Over 2 weeks, animals receiving 10 % of energy as fat developed hepatic steatosis with induction of lipogenic enzymes. Rats fed diets enriched with olive oil (40 % of energy) or menhaden oil (40 % of energy) demonstrated reduced expression of SREBP-1c, reduced hepatic triglyceride content, decreased lipogenesis (i.e. FAS, ACC), and increased expression of enzymes mediating fatty acid oxidation. In addition to the above studies with olive oil, oleic acid and oleic acid rich diets have been shown to decrease hepatic lipogenesis, expression of hepatic lipogenic genes (SREBP1c, SREBP2, FAS, ACC), and expression of hepatic pro-inflammatory genes (tumor necrosis factor alpha, cyclooxygenase-2) [26, 44]. The olive oil-based lipid emulsion used in this study contains refined olive oil and does not contain many of the phenolic compounds (i.e. phenolic alcohols, phenolic acids, flavonoids, lignans, secoiridoids) found in natural olive oil such as oleuropein, tyrosol, or hydroxytyrosol. These phenolic compounds have been shown to reduce hepatic steatosis [40, 45].

Saturated but not unsaturated fatty acids may induce lipotoxicity [46–48], which could contribute to hepatic damage during NAFLD. The effects of saturated fatty acids may depend upon the presence of unsaturated fatty acids. Monounsaturated fatty acids, added to saturated fatty acids, have been reported to attenuate cellular apoptosis and insulin resistance induced by saturated fatty acids [21, 46, 48]. Toll-like receptors recognize saturated fatty acids and may mediate many of their detrimental effects upon tissues [4, 8]. These effects are inhibited by unsaturated fatty acids. Thus, mixtures of different classes of fatty acids may produce effects that differ from those of the individual fatty acids and unsaturated fatty acids may attenuate or block effects of saturated fatty acids.

Based upon the known effects of carbohydrates and fatty acids upon lipogenesis/hepatic steatosis and the results of this study, we hypothesize that lipid mixtures with high unsaturated to saturated fatty acid ratios inhibited de novo lipogenesis induced by sucrose/fructose. By suppressing lipogenesis, the lipid mixtures inhibited development of hepatic steatosis.

### Other considerations

Different fatty acids, despite being structurally similar and belonging to the same chemical class, may elicit different biological effects [49]. Thus, generalizing fatty acid effects by the degree of unsaturation, configuration of the double bonds (i.e. cis or trans), length of the carbon chain, or position of the double bonds (omega-3, omega-6, omega-7, omega-9) may not predict physiological responses. Poudyal et al. [49] has proposed classifying fatty acids based upon biological activity (i.e. neutral, reduce risk factors, increase risk factors, controversial evidence for classification, inadequate research to allow classification). Although such a system may be beneficial, biological effects vary with the model used, organ investigated, parameter measured, underlying illness or inflammatory state, diet utilized, concomitant fatty acids, and many other variables. In addition, lipids used in studies may be complex and contain variable amounts of different fatty acids. It is difficult to classify fatty acids and complex fatty acid mixtures with a large number a different physiologic effects using a single classification system. Despite lack of a biological classification system, it is important not to generalize the effects from one fatty acid to a class of fatty acids. In this study, the predominant saturated fatty acids of the lipid emulsions were palmitic acid and stearic acid. The predominant unsaturated fatty acids varied with the emulsion (Table 1). Soybean oil contained predominantly linoleic acid (with lesser amounts of oleic acid), the olive/soybean oil contained predominantly oleic acid (with lesser amounts of linoleic acid), the macadamia nut oil contained predominantly oleic acid (with lesser amounts of palmitoleic acid), and the fish oil contained predominantly eicosapentaenoic and docosahexaenoic acids. Oils which vary significantly from the study lipids may not produce similar effects.

Most studies of lipogenesis/steatosis utilized a single carbohydrate diet based upon glucose or glucose/sucrose. Few studies have utilized fructose, a common sweetener implicated in the pathogenesis of NAFLD. We utilized three carbohydrate diets (i.e. starch, sucrose, and fructose) so that we could evaluate the interaction between carbohydrate and lipid. It is important to use a starch based diet as a control diet in order to determine the effects of lipids upon normal carbohydrate intake. Most studies have evaluated a limited number of lipid formulations containing high omega-3 or omega-6 polyunsaturated fatty acids or evaluated single fatty acids or triglycerides containing one fatty acid. It is important to use dietary oils since these represent the normal intake of lipids in the diet. There is limited information related to monounsaturated fatty acids and a lack of studies evaluating the effects of oils containing high monounsaturated fatty acids. Many studies utilized very high saturated fatty acid diets, high fat diets, or high calorie diets that are not representative of normal intakes or utilized genetic or nutrient deficient animal models of steatosis. Due to these concerns, we used a normal mouse model, 3 different carbohydrate diets (starch, sucrose, fructose), 4 different lipid formulations derived from dietary oils, a eucaloric normal rodent diet, and normal total and saturated fat intake. The duration of this study is shorter than many published studies of NAFLD. Importantly, the short duration of the study was adequate for inducing hepatic steatosis and was effective at demonstrating the steatosis inhibiting effects of the various lipid mixtures. Thus, we believe that the animal model utilized in this study represents a valid model for studying NAFLD. Further studies are needed to assess the efficacy of specified diets for prevention and treatment of NAFLD. These studies should evaluate interactions between dietary components such as carbohydrates and fats. Future studies should evaluate lipid mixtures with different unsaturated/saturated fatty acid ratios, different levels of lipid and calorie intakes, and potential molecular mechanisms for the effects (i.e. expression of fatty acid synthesis and degradative enzymes and transcription factors, proinflammatory mediators, antioxidant systems).

## Conclusion

In conclusion, this study indicates that both carbohydrate source and lipid intake are important dietary components modulating the development of hepatic steatosis. Simple sugars (fructose > sucrose) are more potent for induction of steatosis than complex carbohydrates. Moderate intake of complex lipids with high unsaturated to saturated fatty acid ratios can inhibit the development of high sucrose/fructose-induced hepatic steatosis. We found no difference in the inhibitory effects of complex lipid formulations based upon polyunsaturated fatty acid (n-3 or n-6) or monounsaturated fatty acid (n-7 or n-9) content. Our results suggest that the total unsaturated fatty acid content is more important than the polyunsaturated fatty acid content of formulations for inhibition of steatosis.
